# Full robotic versus open ALPPS: a bi-institutional comparison of perioperative outcomes

**DOI:** 10.1007/s00464-024-10804-z

**Published:** 2024-05-02

**Authors:** Cristiano Guidetti, Philip C. Müller, Paolo Magistri, Jan Philipp Jonas, Roberta Odorizzi, Philipp Kron, Gianpiero Guerrini, Christian E. Oberkofler, Stefano Di Sandro, Pierre-Alain Clavien, Henrik Petrowsky, Fabrizio Di Benedetto

**Affiliations:** 1 Hepato-Pancreato-Biliary Surgery and Liver Transplantation Unit, University Hospital of Modena “Policlinico”, University of Modena and Reggio Emilia, 41124 Modena, Italy; 2 Swiss HPB and Transplantation Center, Department of Surgery and Transplantation, University Hospital Zurich, Zurich, Switzerland; 3 Department of Surgery, Clarunis – University Centre for Gastrointestinal and Hepatopancreatobiliary Diseases, Basel, Switzerland; 4 Vivévis - Clinic Hirslanden Zurich, Zurich, Switzerland

**Keywords:** Robotic liver surgery, Robotic hepatectomy, Two-stage hepatectomy, Hypertrophy, ALPPS

## Abstract

**Background:**

In primarily unresectable liver tumors, ALPPS (*Associating Liver Partition and Portal Vein Ligation for Staged hepatectomy*) may offer curative two-stage hepatectomy trough a fast and extensive hypertrophy. However, concerns have been raised about the invasiveness of the procedure. Full robotic ALPPS has the potential to reduce the postoperative morbidity trough a less invasive access. The aim of this study was to compare the perioperative outcomes of open and full robotic ALPPS.

**Methods:**

The bicentric study included open ALPPS cases from the University Hospital Zurich, Switzerland and robotic ALPPS cases from the University of Modena and Reggio Emilia, Italy from 01/2015 to 07/2022. Main outcomes were intraoperative parameters and overall complications.

**Results:**

Open and full robotic ALPPS were performed in 36 and 7 cases. Robotic ALPPS was associated with less blood loss after both stages (418 ± 237 ml vs. 319 ± 197 ml; *P* = 0.04 and 631 ± 354 ml vs. 258 ± 53 ml; *P* = 0.01) as well as a higher rate of interstage discharge (86% vs. 37%; *P* = 0.02). OT was longer with robotic ALPPS after both stages (371 ± 70 min vs. 449 ± 81 min; *P* = 0.01 and 282 ± 87 min vs. 373 ± 90 min; *P* = 0.02). After ALPPS stage 2, there was no difference for overall complications (86% vs. 86%; *P* = 1.00) and major complications (43% vs. 39%; *P* = 0.86). The total length of hospital stay was similar (23 ± 17 days vs. 26 ± 13; *P* = 0.56).

**Conclusion:**

Robotic ALPPS was safely implemented and showed potential for improved perioperative outcomes compared to open ALPPS in an experienced robotic center. The robotic approach might bring the perioperative risk profile of ALPPS closer to interventional techniques of portal vein embolization/liver venous deprivation.

**Supplementary Information:**

The online version contains supplementary material available at 10.1007/s00464-024-10804-z.

The need of inducing future liver remnant (FLR) hypertrophy to prevent post-hepatectomy liver failure (PHLF) and adequate oncologic outcomes after liver resection is a key point in dealing with bilobar or large unilobar primary and secondary liver cancers [1–3]. The two-staged hepatectomy called ALPPS (*Associating Liver Partition and Portal Vein Ligation for Staged hepatectomy*) offers fast and extensive hypertrophy of the FLR after portal vein occlusion and in situ splitting of the liver in a first stage, followed by completion hepatectomy one or 2 weeks later [4, 5]. In patients with primarily unresectable liver tumors, ALPPS may offer curative surgery. Compared to PVE, the proposed advantages of ALPPS are a faster growth resulting in a higher resectability rate and therefore, preventing progression of cancer in the interstage interval.

However, despite initial enthusiasm, concerns have been raised about the invasiveness of the procedure represented by a severe morbidity of up to 60% and high mortality rates of 10–20% [6–9].

Recently, robotic liver surgery has gained wide acceptance in specialized hepato-biliary (HPB) centers due to its minimally invasive access with several intraoperative advantages such as enhanced 3D vision with stability and full control of the operative field by the console surgeon and instruments with seven degrees of freedom, which translates to a faster postoperative recovery and less postoperative morbidity. [10, 11] Yet, full robotic ALPPS is a highly demanding procedure that has only been reported in some case reports [12, 13]. The aim of the present bicentric study was to compare a case series of full robotic with open ALPPS in terms of short-term perioperative outcomes.

## Methods

### Study design

**Fig. 1:**
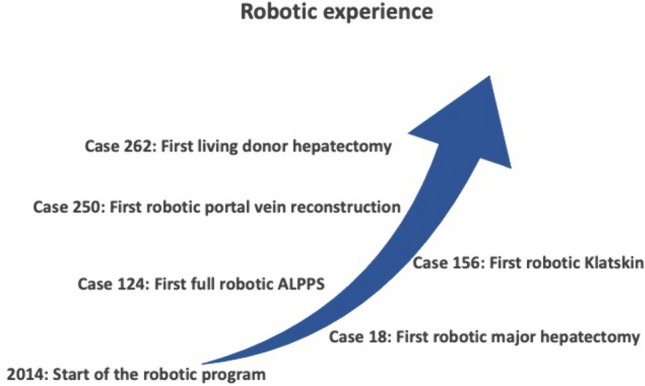
Evolution of the robotic hepato-pancreatobiliary program at the HPB and Liver Transplant Unit of the University of Modena and Reggio Emilia

This retrospective bicentric study was conducted at two high-volume referral centers for HPB surgery between January 2015 and July 2022. Cases for open ALPPS were included from the University hospital Zurich, Switzerland and full robotic ALPPS cases were included from the HPB and liver transplant unit of the University of Modena and Reggio Emilia, Italy. Data were extracted from a prospectively maintained database, the international ALPPS registry. The ALPPS registry is maintained by the Department of Surgery, University of Zurich, Switzerland, approved by the Cantonal Ethics Committee of Zurich (KEK 2013–0326) and is registered at ClinicalTrials.gov (NCT01924741). All patients provided written informed consent. Included were consecutive adult patients with any indication for ALPPS after discussion of the clinical, laboratory and radiology investigations in a multidisciplinary tumor board. After 5 years of robotic HPB surgery and an initial experience of 123 cases of robotic liver resection, robotic ALPPS was started in 2019 (Fig. 1). Since robotic ALPPS was introduced, no more cases of open ALPPS were performed at the HPB and liver transplant unit of the University of Modena and Reggio Emilia. Tumor entity, tumor location, proximity to major vascular structures, and previous abdominal surgery were not considered an exclusion criterion for the robotic approach. Exclusion criteria for the robotic approach were anticipated major vascular resection and reconstruction.

### Perioperative assessment

Preoperative and interstage volumetric assessment was done in collaboration with the radiology departments. All patients underwent preoperative volumetric analyses on the basis of the computed tomography (CT) scan or magnetic resonance imaging (MRI). Volume of the tumor lesions were excluded from the total liver volume (TLV). Estimated total liver volume (eTLV) was calculated using the Vauthey formula [14]. The two parameters used to evaluate necessity of inducing FLR hypertrophy were FLR/eTLV and FLR to body-weight ratio (BWR). After stage 1, FLR hypertrophy was routinely assessed after 5 and 10 days. If hypertrophy was inadequate re-assessment was done every 7–10 days. The aim was to reach at least a FLR/ eTLV of 30% in case of normal liver function tests. In cases of abnormal liver function tests, this threshold was elevated to 35%–40%. Completion of ALPPS stage 2 was always decided after multidisciplinary evaluation of FLR hypertrophy, liver function tests, and general status of the patient.

### Outcome assessment

#### Primary outcome

The main outcome were major complications after both ALPPS stage 1 and 2 defined as grade 3 or higher according to the Clavien–Dindo classification [15].

#### Secondary outcomes

Secondary outcomes were operative time, estimated blood loss (EBL), liver growth rate, post-hepatectomy liver failure (PHLF) according to the International Study Group of Liver Surgery [16], postoperative complications classified according to the Clavien–Dindo classification [15], and the comprehensive complication index (CCI) [17]. The CCI represents an index to assess the cumulative postoperative morbidity. This novel metric measures the overall morbidity on a scale from 0.

(no complication) to 100 (death) [17, 18]. Furthermore, ALPPS completion rate, R0 resection rate, ICU, and hospital stay were assessed.

### Surgical technique of robotic ALPPS (Supplementary video 1)

#### Stage 1

The robotic platforms used for the cases performed was the da Vinci Xi Surgical System (Intuitive Surgical Inc., Sunnyvale, California, United States). Patients were put in supine position, 10–15° anti-Trendelenburg, and 5–10° tilt left. Access to abdominal cavity was obtained through open laparoscopy on the left side of the umbilical scar, introducing the trocar of the AirSeal™ system. Four robotic, 8-mm trocars were placed from right to left hypochondria in a straight line, with a minimum of 8 cm distance in between. Pneumoperiotneum pressure was kept at 12 mmHg. An additional 12-mm trocar was introduced under vision in the right flank. Intraoperative ultrasound (IOUS) was used as needed to identify vascular structure and define relations between lesions and major vessels. After cholecystectomy, hilar dissection was performed until the right hepatic artery and portal vein were encircled with vessel loops.

**Fig. 2:**
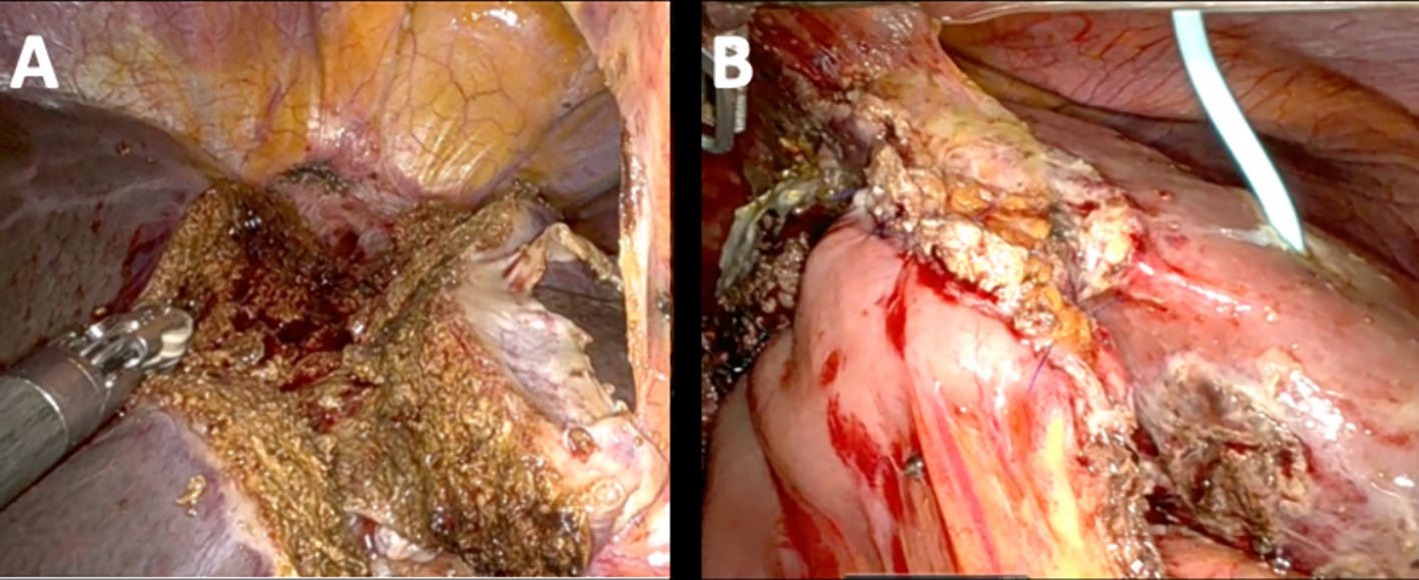
**A**. Intraoperative situs after completion of ALPPS step 1 with the partial transection. **B**. Intraoperative situs after extended right hemihepatectomy with biliary reconstruction at the end of ALPPS step 2

Infrared visualization was used to enhance ICG fluorescence and visualize bile ducts. ICG was routinely administrated 12 h prior to the procedure with a dose of 0.25 mg/kg. Then the right portal vein was divided between hem-o-lok clips or with a stapling device. Liver transection was performed with the harmonic scalpel, bipolar and monopolar currents aiming for complete or near-complete division (Fig. 2A).

Hem-o-lok, titanium clips, ligatures, and sutures were used to achieve division of vasculo-biliary structures within the parenchyma and to obtain hemostasis and biliostasis. At the end of the transection a drain was usually left in between the cut surface.

#### Stage 2

After adequate hypertrophy (see above) stage 2 started using the same incisions as of the stage 1. Adhesiolysis was conducted. Full right mobilization was then performed. The right hepatic artery was divided between clips, then the hilar plate was divided, and both the right and middle hepatic vein were divided with a stapling device. If necessary, biliary reconstruction was performed (Fig. 2B). The specimen was extracted through a Pfannenstiel incision.

### Statistics

Continuous variables were compared with Student’s *t* test and Mann–Whitne test as appropriate. Differences between proportions derived from categorical data were compared with chi-square or Fisher’s exact test. The software used is STATA 15.

## Results

During the study period, 36 patients underwent open- and 7 full robotic ALPPS. Patients in the open group had a higher body mass index (27 ± 4 vs. 23 ± 4 kg/m^2^; *P* = 0.05) and open ALPPS was mainly performed for CRLM (89% vs. 29%), whereas robotic ALPPS was more often performed for cholangiocarcinoma (57% vs. 3%; *P* < 0.01). Other baseline characteristics such as age, sex, American Society of Anesthesiologists score, comorbidities, and model for end-stage liver disease score were similar. Before stage 1, both TLV (1655 ± 282 ml vs. 1398 ± 223 ml; *P* = 0.02) and FLR (495 ± 177 ml vs. 340 ± 99 ml; *P* = 0.03) were higher in the open ALPPS group, resulting in a higher FLR/eTLV (30 ± 10% vs. 25 ± 6%; *P* = 0.16). Preoperative functional liver test with ICG testing were comparable (Table 1).

**Table 1:** Patient characteristics

	Open (*n* = 36)	Robotic (*n* = 7)	*P*
Male sex	24 (67)	5 (71)	0.80
Age, yr	62 ± 11	58 ± 11	0.45
BMI, kg/m^2^	27 ± 4	23 ± 4	0.05
ASA score			0.07
1	0 (0)	1 (14)	
2	17 (47)	4 (57)	
3	19 (53)	2 (29)	
Comorbidities			
Cardiovascular	4 (11)	0 (0)	0.35
Chronic kidney disease	0 (0)	0 (0)	1
Diabetes	6 (17)	3 (43)	0.12
CAPRA-Score^a^	4.7 ± 1.6	5.5 ± 1.5	0.22
Indication			< 0.01
HCC	3 (8)	1 (14)	
iCCA	1 (3)	3 (43)	
pCCA	0 (0)	1 (14)	
CRLM	32 (89)	2 (29)	
Pre-operative lab			
Platelets (× 10^3^/mm^3^)	240 ± 90	225 ± 43	0.67
INR	1.05 ± 0.1	1.02 ± 0.1	0.36
Total bilirubin (mg/dl)	0.5 ± 0.2	2.1 ± 3.8	0.01
MELD	7 ± 0	8 ± 1	0.09
ICG testing pre-op			
PDR	20 ± 5	21 ± 2	0.83
R15	7 ± 6	4 ± 3	0.59
TLV, cc	1655 ± 282	1398 ± 223	0.02
FLR, cc	495 ± 177	340 ± 99	0.03
FLR/eTLV, %	30 ± 10	25 ± 6	0.16
FLR-BWR	0.63 ± 0.22	0.53 ± 0.13	0.22

Data are given as n (%) and median (IQR)

*ASA* American Society of Anesthesiologists, *BMI* body mass index, *BWR* body-weight ratio, *CRLM* colorectal liver metastasis, *FLR* future liver remnant, *HCC* hepatocellular carcinoma, *iCCA* intrahepatic cholangiocarcinoma, *ICG* indocyanine green, *INR* international normalized ratio, *MELD* model for end-stage liver disease, *pCCA* perihilar cholangiocarcinoma, *TLV* total liver volume

^a^Refers to Capobianco et al

### Intraoperative outcomes

In both groups, most patients underwent right trisectionectomy (61% vs. 71%; *P* = 0.69). EBL after both stage 1 (418 ± 237 ml vs. 319 ± 197 ml; *P* = 0.04) and stage 2 (631 ± 354 ml vs. 258 ± 53 ml; *P* = 0.01) was less in the robotic ALPPS group. On the other hand, OT was longer in the robotic ALPPS group after both stage 1 (371 ± 70 min vs. 449 ± 81 min; *P* = 0.01) and stage 2 (282 ± 87 min vs. 373 ± 90 min; *P* = 0.02). Details are shown in Table 2.

**Table 2:** Intraoperative outcomes

	Open (*n* = 36)	Robotic (*n* = 7)	*P*
ALPPS step 1			
EBL, ml	418 ± 237	319 ± 197	0.04
Operation time, minutes	371 ± 70	449 ± 81	0.01
ALPPS step 2			
EBL, ml	631 ± 354	258 ± 153	0.01
Operation time, minutes	282 ± 87	373 ± 90	0.02
Surgical procedure			0.69
Right hemihepatectomy	14 (39)	2 (29)	
Right trisectionectomy	22 (61)	5 (71)	
Liver growth			
FLR pre-stage 2, cc	715 ± 184	584 ± 71	0.07
FLR change, %	53 ± 39	82 ± 44	0.08
FLR change, cc	210 ± 105	245 ± 104	0.08
FLR/eTLV, %	44 ± 11	42 ± 6	0.74
FLR-BWR	0.92 ± 0.23	0.92 ± 0.14	0.94

Data are given as *n* (%) and median (IQR)

*ALPPS* Associating Liver Partition and Portal Vein Ligation for Staged Hepatectomy, *BWR* body-weight ratio, *EBL* estimated blood loss, *FLR* future liver remnant, *TLV* total liver volume

### Post-operative outcomes

After ALPPS stage 1, there was no difference for overall complications (50% vs. 57%; *P* = 1.00) as well as major complications (8% vs. 14%; *P* = 0.62). In line with those results, the cumulative postoperative morbidity assessed by the CCI after stage 1 was not different (12 ± 19 vs. 13 ± 15; *P* = 0.83). There was no difference in terms of occurrence of PHLF after stage 1. One fatality was recorded in the open group after ALPPS stage 1 due to sepsis and multi-organ failure in the setting of PHLF grade C.

There was a higher rate of interstage discharge in the robotic group (37% vs. 86%; *P* = 0.02). Both groups demonstrated adequate FLR increase (53 ± 39% vs. 82 ± 44%; *P* = 0.08) in a similar amount of time (24 days vs. 16 days; *P* = 0.66). Completion of the ALPPS procedure was possible in most cases (97% vs. 100%; *P* = 0.65).

Again, after ALPPS stage 2, there was no difference for overall complications (86% vs. 86%; *P* = 1.00) as well as major complications (39% vs. 43%; *P* = 0.86). The CCI after stage 2 was not different (30 ± 23 vs. 27 ± 12; *P* = 0.79). There was no difference in terms of PHLF after stage 2. After ALPPS stage 2, two casualties occurred in the open cohort, one due to PHLF grade C and the other due to intraoperative myocardial infarction immediately after laparotomy. There was no difference in R0 resection rate (91% vs. 100%; *P* = 0.72), total length of ICU stay (3 ± 2 days vs. 2 ± 2 days; *P* = 0.48) nor total length of hospital stay (26 ± 13 days vs. 23 ± 17 days; *P* = 0.56). Post-operative outcomes are reported in Table 3.

**Table 3:** Post-operative outcome

	Open (*n* = 36)	Robotic (*n* = 7)	*P*
ALPPS step 1 complications^a^			0.62
Minor (I–II)	15 (42)	3 (43)	
Major (IIIa–V)	3 (8)	1 (14)	
Liver failure^c^ after step 1			0.10
*A*	2 (6)	1 (14)	
*B*	0 (0)	1 (14)	
*C*	1 (3)	0 (0)	
ALPPS step 1 CCI^b^	12 ± 19	13 ± 15	0.83
ALPPS step 1 mortality	1 (3)	0 (0)	0.65
Interstage discharge	13 (37)	6 (86)	0.02
Interstage interval, d	24 ± 11	16 ± 7	0.66
Step 2 completion	35 (97)	7 (100)	0.65
ALPPS step 2 complications°			0.86
Minor (I–II)	17 (47)	3 (43)	
Major (IIIa–V)	14 (39)	3 (43)	
Liver failure^c^ after step 2			0.41
*A*	12 (35)	3 (43)	
* B*	3 (9)	2 (29)	
* C*	1 (3)	0 (0)	
ALPPS step 2 CCI^b^	30 ± 23	27 ± 12	0.79
ALPPS step 2 mortality	2 (6)	0 (0)	0.65
ICU stay, d	3 ± 2	2 ± 2	0.48
Hospital stay, d	26 ± 13	23 ± 17	0.56
R0 resection^d^	32 (91)	7 (100)	0.72

Data are given as *n* (%) and median (IQR)

*ALPPS* Associating Liver Partition and Portal Vein Ligation for Staged Hepatectomy, *CCI* comprehensive complication index, *ICU* intensive care unit

^a^Refers to Dindo et al

^b^Refers to Slankamenac et al

^c^Refers to Rahbari et al

^d^Of completed step 2 cases

## Discussion

This is the first study comparing a case series of full robotic with open ALPPS. The analysis shows that robotic ALPPS offers distinct advantages over the open approach, such as reduced blood loss during both ALPPS stages as well as a higher rate of interstage discharge. Even though little selection criteria were applied from the start of the robotic ALPPS program and the results therefore include the learning curve of an experienced robotic HPB center, the surgical morbidity and mortality were comparable for the robotic and open approach. Except for a longer operation time of the robotic approach, robotic ALPPS was associated with similar postoperative outcomes, while no conversion to an open approach was necessary.

Patient safety is of utmost importance when introducing a novel surgical approach. Both the similar major morbidity rate and a lower mortality rate in the robotic group (0% vs. 8%) demonstrate the safe implementation of the robotic ALPPS approach. Most important, no complications were directly attributable to the robotic approach. Of note, robotic ALPPS was implemented after an institutional robotic HPB learning period of 5 years with a previous institutional experience of 123 HPB procedures, including major robotic liver resections and living kidney donation, demonstrating the broad experience with robotic surgery before ALPPS [19].

In a large Italian registry study, open and laparoscopic ALPPS were compared and the minimally invasive approach decreased the overall complication rate after stage 1, lowered the PHLF rate, and was even associated with a reduced in-hospital mortality rate in multivariate analysis [20]. A similar or even more pronounced effect could be expected for the robotic ALPPS approach after surpassing the learning curve of around 20–60 cases for major liver resections [11, 21]. Both centers included in this study have not implemented laparoscopic ALPPS and therefore, a direct comparison to laparoscopic ALPPS is missing. However, due to an improved 3D vision with magnification of the operative field and enhanced versatility of the robotic instruments the robotic approach offers several potential benefits as already demonstrated in hepato-biliary surgery [22, 23].

While in the current study no cases had to be converted to an open approach, the main contributing factors for a significant conversion rate were adhesions and bleeding especially in the technically more challenging stage 2 (Stage 1: 4% and stage 2: 28%) [20]. The second stage of ALPPS, which typically includes an extensive adhesiolysis, followed by a right or extended right hemihepatectomy is technically much more complex. In the Italian registry study, after minimally invasive stage one, two-third of patients were operated with a laparoscopic approach showing the growing interest of surgeons in minimizing the operative trauma for the patient. Irrespective of the approach for stage 2, minimally invasive stage 1 decreased the overall surgical impact on patients as demonstrated by a lower overall complication rate and in line with this finding, a higher interstage discharge rate. In our own experience, patients requiring complex vasculo-biliary resections and reconstructions, therefore undergoing robotic ALPPS stage 1 with open stage 2, equally benefitted from the reduced surgical trauma as demonstrated by a low postoperative morbidity.

In the current study, the liver regeneration was not affected by the surgical approach. Both the open and robotic approach showed adequate liver hypertrophy and a similar PHLF rate. These findings are somehow contradictory to the two largest series of minimally invasive, laparoscopic ALPPS, where the minimally invasive approach was associated with significantly less clinically relevant PHLF in both studies [20, 24]. While the sample size of the current study does not allow a statistically sound comparison, it has to be kept in mind, that the FLR/eTLV rate was considerably lower in the robotic group before stage 1 (25% vs. 30%), a factor that might have mitigated beneficial effects of the robotic approach on PHLF rate.

The study is certainly limited by the sample size of the robotic group, limiting statistical comparisons. However, currently the literature consists of some single-case reports, therefore, this study represents the largest consecutive series of full robotic ALPPS. The limited application of the robotic ALPPS approach introduces potential bias with regard to patient selection and uncontrolled baseline differences of the groups. Confounders such as different ALPPS indications (CRLM vs. primary liver tumors) and differences in the FLR may have further affected the outcomes of this study. Furthermore, the cost of the two approaches was not directly compared. However, several studies on robotic hepato-pancreato-biliary surgery showed that the robotic instruments are more expensive. If patients experience less complications and therefore have a shorter hospital stay, the robotic approach is cost effective. [25–27] Given the higher rate of interstage discharge and a 3-day difference in length of stay, we assume cost effectiveness of the robotic approach even with the longer operation time in this cohort.

## Conclusion

In a highly experienced robotic HPB center, full robotic ALPPS was safely implemented and showed potential for improved perioperative outcomes compared to open ALPPS including a higher rate of interstage discharge potentially a surrogate for a lower surgical trauma. The robotic approach might help to transform the ALPPS method into a less invasive technique, bringing its perioperative risk profile closer to the interventional techniques of portal vein embolization and liver venous deprivation while allowing for a fast and extensive FLR hypertrophy.

### Supplementary Information

Below is the link to the electronic supplementary material.

Supplementary file1 (MP4 313369 KB)
